# Gestational diabetes mellitus as a risk factor for future Type-2 diabetes mellitus: An experience from a tertiary care diabetes hospital, Karachi - Pakistan

**DOI:** 10.12669/pjms.40.5.7507

**Published:** 2024

**Authors:** Musarrat Riaz, Nazish Waris, Akifa Saadat, Asher Fawwad, Abdul Basit

**Affiliations:** 1 Musarrat Riaz, FCPS. Associate Professor, Department of Medicine, Consultant Endocrinologist, Baqai Institute of Diabetology and Endocrinology, Baqai Medical University, Karachi, Pakistan; 2 Nazish Waris, Ph.D. Senior Lecturer, Department of Biochemistry, Research Associate, Baqai Institute of Diabetology and Endocrinology, Baqai Medical University, Karachi, Pakistan; 3 Akifa Saadat, B.S. Research Officer, Research Department, Baqai Institute of Diabetology and Endocrinology, Baqai Medical University, Karachi, Pakistan; 4 Asher Fawwad, Ph.D. Professor & Head of the Biochemistry Department, Baqai Institute of Diabetology and Endocrinology, Baqai Medical University, Karachi, Pakistan; 5 Abdul Basit, FRCP. Professor of Medicine, Baqai Institute of Diabetology and Endocrinology, Baqai Medical University, Karachi, Pakistan

**Keywords:** GDM, T2DM, Duration, Risk factors

## Abstract

**Objective:**

To evaluate the history of gestational diabetes mellitus and other risk factors in women presenting with Type-2 diabetes mellitus at a tertiary care hospital.

**Methods:**

This cross-sectional study was carried out at Baqai Institute of Diabetology & Endocrinology (BIDE), Baqai Medical University (BMU), Karachi-Pakistan from July 2019 to May 2022. Women with Type-2 diabetes mellitus (T2DM) visiting outpatient department of BIDE with a previous history of GDM were recruited. Details were obtained on pre-designed questionnaire after taking informed written consent.

**Results:**

A total of 378 women who had a prior history of GDM were included. Mean age (years) was 43.53±10.17. Mostly women were obese (BMI = 30.53±6.08) and have sedentary lifestyle. Mean HbA1c (%) was 9.08±2.24. This study found family history of T2DM and hypertension were common risk factors in women with GDM history. Mostly, women were diagnosed as GDM during 2^nd^ trimester 153(42%) and was mainly seen in multiparous women (occur in 4^th^ and above pregnancy). We found hypertension as common complication during pregnancy. Around 46% women developed T2DM within one year of GDM diagnosis, and 29.6% between one to five years.

**Conclusion:**

Majority of women with GDM developed T2DM within five years of diagnosis. The potential associated risk factors were age, family history of diabetes, insulin use during pregnancy, trimester of GDM diagnosis, and hypertension during pregnancy. Awareness and life style modifications along with regular post-partum follow up with screening for T2DM should be part of GDM management to prevent or delay the occurrence of this serious complication.

## INTRODUCTION

Gestational diabetes mellitus (GDM) is an adverse pregnancy complication that carries unique diabetes-related risks and potential long-term consequences for mother and unborn baby. The approximate annual birth complications with hyperglycemia in pregnancy has been reported as 16%.^1^ GDM has now become a global public health burden that increases the risk of morbidity and mortality, birth traumas, neonatal hypoglycemia, and other severe health problems. It is a significant cause of congenital abnormalities and stillbirths. Annually, stillbirth is estimated to occur in 2.6 million pregnancies worldwide.^2^

It was also reported that the offspring are potentially at a higher risk of developing obesity, adiposity and disorders of glucose metabolism (insulin resistance and type-2 diabetes) later in life. Cesarean births and pregnancy-related hypertension are more common in women with GDM.^3^ T2DM is a rising health issue which reduces the life-expectancy as well as survivors’ quality of life.^4^ Women with GDM are nearly at a 10-fold higher risk of developing T2DM than those with a normoglycemic pregnancy.^5^

A further systematic review evaluated the rate of compliance with screening, and the prevalence of T2DM in Asian women, reporting incidences rate between 2.8% and 58% in women with previous GDM.^6^,^7^ Southeast Asia has the greatest prevalence of GDM, which is estimated to be 24.2%.^8^ In Bangladesh, the frequency has been reported varying from 13.2% to over 40%, and from 3.8 to 22% in different parts of India depending on the geographical location.^9^ In Pakistan, the incidence of GDM varies from 4.2 to 26% depending on the region and the diagnostic techniques used.

GDM correlates with age; the incidence of GDM increases by approximately eight times, with an increase in the rate of women giving birth at a more advanced age. Several risk factors including body mass index, family history of diabetes, hypertension, the decrease in physical activity, and the adoption of modern lifestyles contribute to an increase in the prevalence of GDM.^10^ GDM has serious adverse implications for the health of current and future generations through genetic and environmental mechanisms.^11^ The condition also places a heavy financial burden on healthcare systems. Limited research has been conducted in Pakistan so far despite its alarming consequences. Therefore, this study was conducted to assess the history of gestational diabetes mellitus and other risk factors in women presenting with Type-2 diabetes mellitus at a tertiary care hospital.

## METHODS

This cross-sectional study was conducted at Baqai Institute of Diabetology & Endocrinology (BIDE), a tertiary care hospital of Karachi, Pakistan.

### Ethical Approval:

The study was approved by an Institutional Review Board of Dow University of Health Sciences (DUHS) (IRB- 1413/DUHS/Approval/2019).

### Inclusion Criteria:

Using consecutive sampling technique, all the women who attended the diabetic outpatient department of BIDE from July 2019 to May 2022, with the history of GDM, were included. GDM was considered if the participants visited with any previous medical history reported by their physician and lab report. Current status of the participants with previous history of GDM were obtained on a pre-designed questionnaire through face-to-face interviews by a trained research officer after obtaining a written informed consent. Women having T1DM, T2DM before pregnancy and with T2DM having no previous history of GDM was excluded.

The proforma included the questions about age, educational background, ethnicity, physical activity, duration of diabetes, family history of T2DM and GDM, previous GDM in earlier pregnancies, and current treatment (oral hypoglycemic agents (OHAs) or insulin). Physical activity include exercise as sedentary (no exercise), light (home chores/little exercise), moderate (brisk walk for 30 minutes), and heavy (exercise > 30 minutes). Any complications during pregnancy like hypertension, urinary infection, jaundice etc. or complication during child birth were also investigated. Questions about stillbirth, miscarriage or abortion were also inquired.

Anthropometric measurements such as height and weight were noted. A calibrated and standardized digital scale was used to measure participants’ weight in kilograms (kg). Participants were weighed in light clothing and were also asked to remove shoes and socks. The height was measured with the help of a stadiometer in centimeter (cm). Participants were asked to remove shoes and stand straight in an upright position along their back towards wall. Body Mass Index (BMI) kg/m² was calculated by dividing weight (kg) by squared height (m). BMI was classified as per Asian-Pacific cutoff points as normal 18.5–22.9kg/m^2^, overweight 23.0– 24.9kg/m^2^ and obesity ≥25 kg/m^2^.^12^ Blood pressure was measured with mercury sphygmomanometer. Participants were requested to take 10 minutes rest in a sitting position before measurement of blood pressure to reduce variation. Mean of two readings were used in the study. Blood pressure <140/90mmHg was considered as normal and high blood pressure was considered as blood pressure ≥140/90mmHg.^13^ HbA1c was analyzed by high-performance liquid chromatography method using Bio-Rad D-10. HbA1c <7% was considered best control, and seven and above as uncontrolled glycemic or poor control.^14^

### Statistical Analysis:

Analyses were performed using Statistical Package for Social Sciences (SPSS version 20.0). For continuous variables, statistics included n (number of observation), mean, standard deviation, as well as frequencies and percentages for categorical variables. P-value <0.05 considered as statistically significant.

## RESULTS

A total of 378 women who had a prior history of GDM were included. Current status of baseline characteristics of the study participants is presented in Table-I. Mean age (years) was 43.53±10.17. Among these, 4(1.1%) women were below 25 years, 90(23.9%) were between 26 to 35 years and 283(75.1%) women were ≥ 36 years. Mean BMI was 30.53±6.08(kg/m^2^) and systolic/diastolic blood pressure (mmHg) was 125.75±18.01/78±12.46. Most of the women had sedentary lifestyle. The current status of glycemic index and medical history related to GDM is shown in Table-II. Current status of mean HbA1c (%) was found to be 9.08±2.24 (12.6% were observed with <7% HbA1c and 87.4% were found with ≥ 7% HbA1c). Majority of women were being treated with OHA for type-2 diabetes after GDM. Upon asking previous history it was found that mostly, women were diagnosed as GDM during second trimester (42%) and was mainly seen in multiparous women (occur in fourth and above pregnancy). Around 46.5% women used insulin during pregnancy. Hypertension was the common complication during pregnancy 117(31%).

**Table-I:** Current status of baseline characteristics of study participants.

Parameters	n(%) or Mean ± SD
N	378
Age (years)	43.53±10.17
25 and under	4(1.1%)
26 to 35	90(23.9%)
36 and over	283(75.1%)
BMI (kg/m^2^)	30.53±6.08
Systolic BP (mmHg)	125.75±18.01
Diastolic BP (mmHg)	78±12.46
Ethnicity	
Urdu speak/Muhajir	266(70.4%)
Punjabi	24(6.3%)
Sindhi	18(4.8%)
Pathan	47(12.4%)
Balochi	9(2.4%)
Others	14(3.7%)
Marital status	
Married	357(94.4%)
Divorced/separated	7(1.9%)
Widowed	14(3.7%)
Exercise	
Sedentary	184(48.9%)
Light	128(34%)
Moderate	53(14.1%)
Heavy	11(2.9%)

Data presented as n(%) or Mean ± SD.

**Table-II:** Glycemic Index and medical history of study participants.

Parameters	n(%) or Mean ± SD
N	378
***Current status of the participants***	
HbA1c (%)	9.08±2.24
<7%	32((12.6%)
≥7%	222(87.4%)
***Present, regular treatment of T2DM after GDM***	
None	6(1.6%)
Diet	6(1.6%)
OHA	328(86.8%)
insulin	197(52.1%)
***Previous history of the participants***	
***Trimester at GDM diagnosis***	
1^st^ trimester	71(19.5%)
2^nd^ trimester	153(42%)
3^rd^ trimester	140(38.5%)
***GDM at pregnancy***	
1^st^	43(12.5%)
2^nd^	50(14.6%)
3^rd^	81(23.6%)
4^th^ and above	169(49.3%)
***Used Insulin during pregnancy***	
Yes	164(46.5%)
No	189(53.5%)
***Complications during pregnancy***	
Hypertension	117(31%)
Urinary infection	9(2.4%)
Jaundice	2(0.5%)
Others	17(4.5%)
No	243(64.3%)
***Obstetric History***	
babies alive	367(97.1%)
still births	95(25.1%)
miscarriage	172(45.9%)
surgical abortions	30(7.9%)

Data presented as n (%) or Mean ± SD.

**Fig.1:**
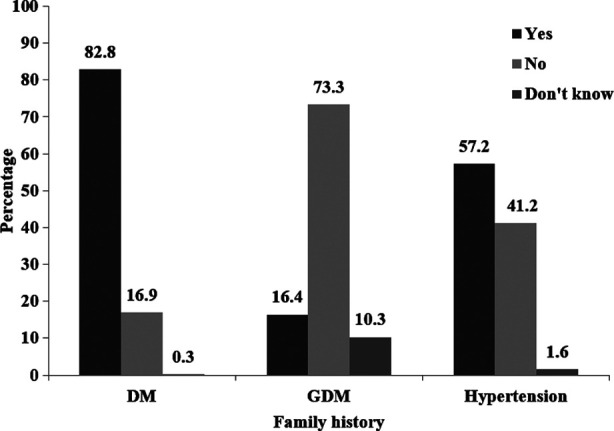
Frequency of associated familial risk factors for GDM DM: diabetes mellitus, GDM: gestational diabetes mellitus.

**Fig.2:**
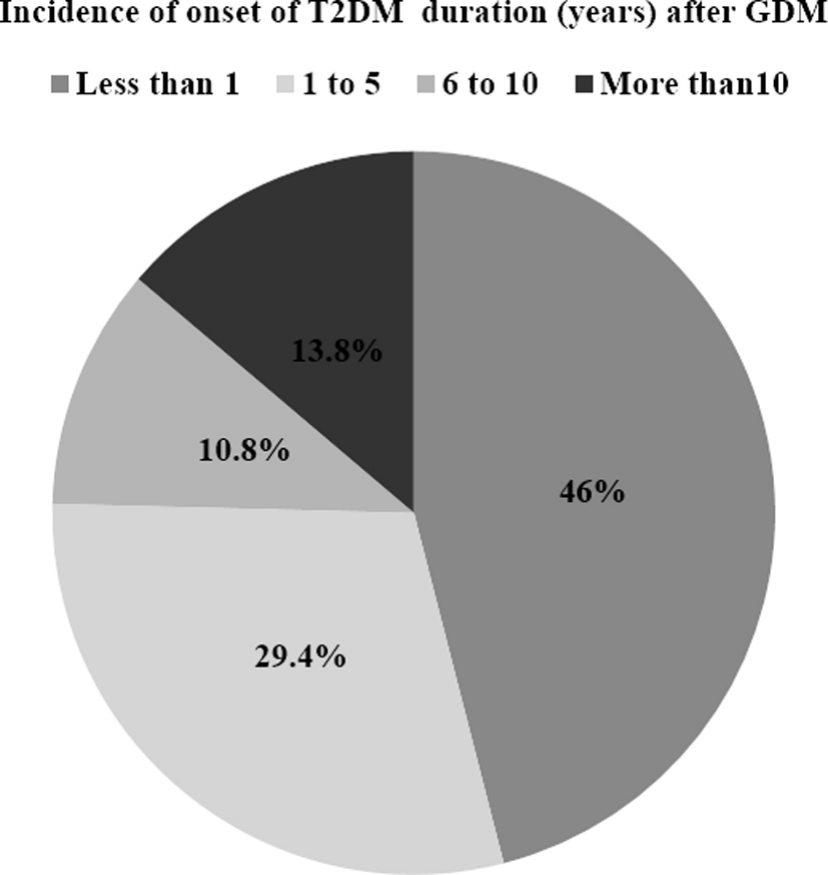
Incidence of onset of T2DM duration (years) after GDM. GDM: gestational diabetes mellitus, T2DM: type-2 diabetes mellitus.

Frequency of potential familial associated risk factors are shown in Fig.1. It was noted that most of the women had family history of T2DM (82.8%) and family history of hypertension (57.2%) as common associated risk factors. Family history of GDM was reported in 16.4% women. The onset of T2DM from GDM is presented in Fig.2. It was found that 46% women developed T2DM within one year of GDM diagnosis, 29.6% between one to five years and 10.8% between six to ten years duration.

Association of potential risk factors of participants with current status and previous history with duration of T2DM from GDM are shown in Table-III. Current status of women who developed T2DM from GDM suggesting that age (p-value <0.0001) and family history of diabetes (p-value = 0.034) are statistically significantly potential associated risk factor, while history of GDM suggesting that insulin use during pregnancy (p-value <0.0001), trimester of GDM diagnosis (p-value=0.03), and hypertension during pregnancy (p-value=0.038) are also the significantly potential associated risk factors in developing T2DM from GDM.

**Table-III:** Association of potential risk factors of participants with duration of T2DM from GDM

Parameters	Less and equal to 5 years	More than 5 years	P-value
n	285	93	-
***Current status of potential risk factors***			
Age (years)			
25 and under	4(1.4%)	0(0%)	<0.0001
26 to 35	81(28.5%)	9(9.7%)	
36 and over	199(70.1%)	84(90.3%)	
***Family history of Diabetes***			
Yes	239(83.9%)	74(79.6%)	0.161
No	46(16.1%)	18(19.4%)	
Do not know	0(0%)	1(1.1%)	
***Family history of GDM***			
Yes	50(17.5%)	12(12.9%)	0.080
No	211(74%)	66(71%)	
Do not know	24(8.4%)	15(16.1%)	
***Family history of Hypertension***			
Yes	158(56.2%)	56(60.2%)	0.670
No	119(42.3%)	35(37.6%)	
Do not know	4(1.4%)	2(2.2%)	
***Body mass index (kg/m^2^)***			
<25	50(18.2%)	12(13.2%)	0.261
≥25	224(81.8%)	79(86.8%)	
***Blood pressure (mmHg)***			
<140/90	185(67%)	59(64.8%)	0.701
≥140/90	91(33%)	32(35.2%)	
***HbA1c (%)***			
<7	22(11.3%)	10(16.9%)	0.214
7 to 9	87(44.6%)	30(50.8%)	
10 or above	86(44.1%)	19(32.2%)	
***Previous history of potential risk factor***			
***Used insulin during pregnancy***			
yes	147(55.7%)	17(19.1%)	<0.0001
No	117(44.3%)	72(80.9%)	
Complications during pregnancy			
Hypertension	92(32.3%)	25(26.9%)	0.038
Urinary infection	9(3.2%)	0(0%)	0.083
Jaundice	2(0.7%)	0(0%)	0.418
Others	11(3.9%)	6(6.5%)	0.295
None	180(63.2%)	63(67.7%)	0.423
***Trimester at GDM diagnosis***			
1st trimester	62(22.6%)	9(10%)	0.03
2nd trimester	112(40.9%)	41(45.6%)	
3rd trimester	100(36.5%)	40(44.4%)	

Data presented as n(%): P-value <0.05 considered to be statistically significant.

## DISCUSSION

The onset of T2DM from GDM in majority of women was within five years of diagnosis. GDM to T2DM implies a possible complication after postpartum. Early progression of T2DM with GDM history consistent to Bengtson et al. who reported that up to 50% of women with history of GDM progress to T2DM within five years of postpartum.^15^ Our findings are also in agreement with Kim et al. study who showed a higher cumulative incidence of T2DM in the first five postpartum years.^16^

It was earlier studied that genetic and behavioral factors are involved with family history of hypertension and diabetes and in women it may be predisposed to an increased preeclampsia risk.^10^ In this study, we found a family history of T2DM and hypertension are the common associated risk factors in GDM women progressing to T2DM. We also found hypertension as a complication during pregnancy was significantly high in women with below five years duration of diabetes progression. Lee et al reported that women had rising trend of hypertension during pregnancy.^17^ Our results are in consistent with Li et al who found an association between GDM and family history of diabetes mellitus.^18^ Our findings are also in agreement with Wagan et al who reported family history of diabetes mellitus and age as a classic risk factors associated with less than five years duration of GDM progression to T2DM.^19^ Risk of GDM increases with increasing age is in line with Vounzoulaki et al. study.^4^

Similar to previous reported data, insulin use during pregnancy is also the significant potential associated risk factor in women with less duration of GDM onset to T2DM.^20^ Noctor et al. and Dunne et al. also reported that women with use of insulin during pregnancy were more prone of future progression to diabetes and/or abnormal glucose tolerance.^20^ We found most of the women with GDM diagnosis in second trimester and were multiparous women (occur in fourth and above pregnancy). However, Brand et al reported that women who diagnosed with a third trimester were usually older, obese, having hypertensive disorder of pregnancy and were more often multiparous.^21^ According to recent estimates in Pakistan 58% women had generalized obesity and 62.7% had central obesity that have a pivotal role in GDM and T2DM development.^12^,^22^ Literature suggest that after pregnancy 7% increment occur in weight.^23^ We also found most of the women in current state were overweight and or obese that can be significantly associated to an increase in the number of T2DM.^24^ Though, we found non-significant results for BMI with duration of diabetes. However, urgent lifestyle modification strategies to manage this high risk cardio metabolic risk factor are necessary.^25^

It was noted that number of women were not checking blood glucose levels after delivery which may results in late diagnosis of T2DM. So, it is important to regularly monitor blood glucose levels post-partum so that diagnosis of T2DM is not delayed and early management can be started.^26^ Our results also suggest that women who are at high risk should use their worthwhile time window for preventing GDM progression to T2DM and prioritized preventative interventions such as diet, physical activity, breastfeeding and postpartum weight management.^27^ In consequence, free-of-cost GDM screening and postpartum diabetes screening awareness programs are needed and dietary and lifestyle interventions can be a significant step towards diabetes prevention and related complications in this region of the world to overcome the burden of diabetes. Education session regarding importance of breastfeeding and weight management in women with history of GDM should also be the necessary steps.^28^ Future researches are required across heterogeneous populations to evaluate the effectiveness and cost effectiveness of preventive interventions over long term period.

### Strengths and Limitations:

We used a consecutive sampling method; a single centered and recall bias are the limitations. However, reporting duration and long term risk factors of T2DM progression in women with history of GDM in Pakistan is strength of this study.

## CONCLUSION

Majority of women with GDM developed T2DM within five years of diagnosis. The potential associated risk factors were age, family history of diabetes, insulin use during pregnancy, trimester of GDM diagnosis, and hypertension during pregnancy. These findings suggest a need for epidemiological studies in developing countries. Awareness and life style modifications along with regular post-partum follow up with screening for T2DM should be part of GDM management to prevent or delay the occurrence of this serious complication.
